# Surveillance of leishmaniasis cases from 15 European centres, 2014 to 2019: a retrospective analysis

**DOI:** 10.2807/1560-7917.ES.2022.27.4.2002028

**Published:** 2022-01-27

**Authors:** Gert Van der Auwera, Leigh Davidsson, Pierre Buffet, Marie-Thérèse Ruf, Marina Gramiccia, Stefania Varani, Carmen Chicharro, Aldert Bart, Gundel Harms, Peter L. Chiodini, Hanne Brekke, Florence Robert-Gangneux, Sofia Cortes, Jaco J Verweij, Alessandra Scarabello, Sara Karlsson Söbirk, Romain Guéry, Saskia van Henten, Trentina Di Muccio, Elena Carra, Pieter van Thiel, Martin Vandeputte, Valeria Gaspari, Johannes Blum, LeishMan Surveillance network, LeishMan Surveillance Network members who contributed to this article (in addition to authors above), Emmanuel Bottieau, Jan Clerinx, Lieselotte Cnops, José Manuel Cristovão, Jean-Claude Dujardin, Eleonora Fiorentino, Jean-Pierre Gangneux, Luigi Gradoni, Andreas K Lindner, Diana Lockwood, Stephen Walker, Aldo Scalone, Johan van Griensven

**Affiliations:** 1 Institute of Tropical Medicine, Antwerp, Belgium; 2 The Public Health Agency of Sweden, Solna, Sweden; 3 Service des maladies infectieuses et tropicales, AP-HP, Hopital Necker, Paris, France; 4 Swiss Tropical and Public Health Institute, Basel, Switzerland; 5 University of Basel, Basel, Switzerland; 6 Istituto Superiore di Sanità, Rome, Italy; 7 IRCCS Azienda Ospedaliero-Universitaria di Bologna, Bologna, Italy; 8 Department of Experimental, Diagnostic and Specialty Medicine, University of Bologna, Bologna, Italy; 9 Instituto de Salud Carlos III, Madrid, Spain; 10 Amsterdam University Medical Centre, Amsterdam, the Netherlands; 11 Institute of Tropical Medicine and International Health, Charité – Universitätsmedizin Berlin, corporate member of Freie Universität Berlin and Humboldt – Universität zu Berlin, Berlin, Germany; 12 Hospital for Tropical Diseases, London, United Kingdom; 13 Oslo University Hospital, Oslo, Norway; 14 Univ Rennes, CHU Rennes, Inserm, EHESP, Irset - UMR_S 1085, Rennes, France; 15 Global Health and Tropical Medicine, Instituto de Higiene e Medicina Tropical, Universidade NOVA de Lisboa, Lisbon, Portugal; 16 Microvida Laboratory for Medical Microbiology and Immunology, Elisabeth-TweeSteden Hospital, Tilburg, the Netherlands; 17 National Institute for Infectious Diseases ‘Lazzaro Spallanzani’, Rome, Italy; 18 Division of Infection Medicine, Lund University, Lund, Sweden; 19 Hôpital privé du Confluent, Nantes, France; 20 Istituto Zooprofilattico Sperimentale della Lombardia e dell' Emilia-Romagna ‘Bruno Ubertini’, Brescia, Italy; 21 The members of the network are listed under Investigators

**Keywords:** leishmaniasis, *Leishmania*, Europe, surveillance, authochthonous, imported, travel

## Abstract

**Background:**

Surveillance of human leishmaniasis in Europe is mostly limited to country-specific information from autochthonous infections in the southern part. As at the end of 2021, no integrated analysis has been performed for cases seen across centres in different European countries.

**Aim:**

To provide a broad perspective on autochthonous and imported leishmaniasis cases in endemic and non-endemic countries in Europe.

**Methods:**

We retrospectively collected records from cutaneous, mucosal and visceral leishmaniasis cases diagnosed in 15 centres between 2014 and 2019. Centres were located in 11 countries: Belgium, France, Germany, Italy, the Netherlands, Norway, Portugal, Spain, Sweden, Switzerland and the United Kingdom. Data on country of infection, reason for travelling, infecting species, age and sex were analysed.

**Results:**

We obtained diagnostic files from 1,142 cases, of which 76%, 21% and 3% had cutaneous, visceral, and mucosal disease, respectively. Of these, 68% were men, and 32% women, with the median age of 37 years (range: 0–90) at diagnosis. Visceral leishmaniasis was mainly acquired in Europe (88%; 167/190), while cutaneous leishmaniasis was primarily imported from outside Europe (77%; 575/749). Sixty-two percent of cutaneous leishmaniasis cases from outside Europe were from the Old World, and 38% from the New World. Geographic species distribution largely confirmed known epidemiology, with notable exceptions.

**Conclusions:**

Our study confirms previous reports regarding geographic origin, species, and traveller subgroups importing leishmaniasis into Europe. We demonstrate the importance of pooling species typing data from many centres, even from areas where the aetiology is presumably known, to monitor changing epidemiology.

## Introduction

Kinetoplastid parasites of the genus *Leishmania* cause a variety of diseases in humans, collectively known as the leishmaniases [1]. Visceral leishmaniasis (VL) is a systemic parasitic infection characterised by fever, weight loss, anaemia, and hepato- and splenomegaly. If not treated, the disease is generally lethal. Cutaneous leishmaniasis (CL) displays different levels of severity, from single benign self-healing lesions to complex clinical presentations with multiple lesions on different body parts that are often difficult to treat successfully. A complication of CL is mucosal leishmaniasis (ML), affecting mucosal tissues primarily of the nose and mouth, with potential mutilating and stigmatising consequences. ML can manifest either as a primary infection, possibly with concomitant cutaneous lesions, or after a previously resolved cutaneous infection.

Transmission of the parasite occurs through blood-feeding female sandflies. In Europe, the vector mainly occurs in the southern countries, primarily those of the Mediterranean basin. Several papers have described the epidemiology of endemic leishmaniasis in Europe [2,3]. However, analyses on imported leishmaniasis in endemic and non-endemic regions have rarely integrated data from more than a single country [4,5]. *L. infantum* and *L. tropica* are the only species known to be transmitted in Mediterranean Europe, but globally between 15 and 20 *Leishmania* species are pathogenic to humans [6].

The European LeishMan network was established in 2010, with the aim of sharing leishmaniasis case management data, and harmonising diagnosis and treatment [7]. To reveal epidemiological trends of the recent years (2014–19), we analysed case data from 15 of the 33 centres of the network, all situated in western Europe and Scandinavia. To our knowledge, this is the largest analysis of the leishmaniases in Europe to date, thereby contributing to permanent monitoring of autochthonous and travel-related cases in Europe [6,8], and supplementing surveillance efforts in eastern European countries [9,10].

## Methods

### Study setting and period

Fifteen centres within the European LeishMan network from 11 countries in west and north Europe (Table 1) shared their diagnostic leishmaniasis case data from the period 2014–19 in a common database. Because of the geographic spread of the participating centres, it is highly unlikely that the same patient would have visited more than one centre during the study period, minimising the chances of counting the same case twice. For each case, the following variables were collected: (i) year of diagnosis, (ii) age at time of diagnosis, (iii) sex, (iv) type of disease (CL, ML, VL, or a combination of these), (v) autochthonous or imported (including reason for travel), (vi) probable country of infection, (vii) the species, species complex or subgenus of the parasite and (viii) the genomic target and method that were used for parasite typing.

**Table 1:** Participating European centres of the LeishMan network^a^, country of diagnosis and number of leishmaniasis cases, 2014–2019 (n = 15)

Centres	Country of diagnosis	Number of cases (n = 1,142)
Institute of Tropical Medicine Antwerp	Belgium	124
Centre Hospitalier Universitaire de Rennes	France	38
Necker Pasteur Paris	France	128
Charité-Universitätsmedizin Berlin	Germany	64
INMI Lazzaro Spallanzani	Italy	10
Istituto Superiore di Sanità Rome	Italy	113
University Hospital of Bologna	Italy	109
Amsterdam University Medical Centres	Netherlands	86
Elisabeth-TweeSteden Hospital Tilburg	Netherlands	18
Oslo University Hospital	Norway	42
Instituto de Higiene e Medicina tropical	Portugal	22
Instituto de Salud Carlos III	Spain	86
Public Health Agency of Sweden	Sweden	133
Swiss Tropical and Public Health Institute	Switzerland	121
Hospital for Tropical Diseases	United Kingdom	48

INMI: Istituto Nazionale per le Malattie Infettive.

^a^ The European LeishMan network for diagnosis, treatment and surveillance of leishmaniasis in Europe was established in 2010 and currently has 33 affiliated institutes.

### Age, sex and year of diagnosis

Because the exact infection date is unknown, the date of sampling for disease confirmation was used. Age and sex were recorded; the age of the patient on this date was recorded as the age at time of diagnosis, which was analysed in periods of 10 years. As the study uses retrospective data, the travel and disease history was not uniformly recorded across centres, but we aimed to collect the first disease episode for every case. We took utmost care to avoid inclusion of relapses from infections dating before 2014, even though this cannot be guaranteed if the patient visited other clinics before consulting one of the centres participating to the study. For ML, however, some cases are likely a relapse from an earlier CL manifestation, especially those imported from the New World.

### Type of disease 

The three main clinical manifestations of a *Leishmania* infection were discriminated: VL, CL, and ML. A case was interpreted as ML if mucosal tissue was affected, irrespective of concomitant or earlier cutaneous lesions elsewhere on the body. The same case definitions are used throughout the LeishMan network. The definitions of these main leishmaniasis manifestations are straight-forward, and this study did not stratify according particular sub-categories of CL and ML where classification would be more ambiguous.

### Autochthonous versus imported cases 

An autochthonous case was considered as an infection that took place in the country where it was diagnosed. All other cases were considered as imported or travel related, e.g. an infection diagnosed in France but acquired in Spain was considered an imported infection, even though it was acquired in Europe. For imported cases, we further subdivided this category into migrants, tourists, military personnel, and people visiting friends or relatives (VFR). If there was another reason for travelling, or the reason for travelling was not known, the case was categorised as ‘Traveller unspecified’, to make the distinction with an autochthonous case. The classification ‘Unknown’ was reserved for those cases without information distinguishing an imported or autochthonous infection.

### Country of infection

The ‘country of infection’ was defined as the country where the case was most likely infected. Some cases, especially migrants and tourists, often travelled through various endemic regions, so the exact country of infection was impossible to establish. For migrants, we assumed they were infected in their home country unless other countries were mentioned in the diagnostic records. Whenever the exact country of infection was unclear from the medical record, we pragmatically grouped them into the following regions from the Old World: (i) Mediterranean, (ii) Sub-Saharan Africa and (iii) the Middle East including Pakistan, and from the New World: (iv) South America and (v) Middle America (Mexico, Central America and the West Indies). If no information on the source country was available, or patients visited several of the aforementioned regions, the case was categorised as (vi) ‘Unknown’.

### Species, species complex and subgenus

The taxonomy of the *Leishmania* genus as used in this paper is listed in Table 2 [11-13]. Most centres could reliably determine the species complex of the aetiological agent, using a variety of genomic targets and analysis methods. However, typing the exact species within the complex is often more challenging because of the genetic similarity within the complex and/or lack of a clear consensus on species definition [14]. In some cases, the species was determined by a referring centre, in which case the typing method was unknown. When a species was reported, but the method used does not allow reliable discrimination within the complex, only the species complex information was retained. Even though utmost care was taken to ensure correct identification down to the species level, the analysis was primarily limited to the species complex. In some cases, the typing was limited to the subgenus level, only discriminating between *L.* (*Leishmania*) and *L.* (*Viannia*).

**Table 2:** Taxonomy of the *Leishmania* genus

Genus	Subgenus	Complex	Species
*Leishmania*	*L.* (*Leishmania*)	*L. donovani*	*L. donovani*
			*L. infantum* (syn. *L. chagasi* in New World)
		*L. major*	*L. major*
		*L. mexicana*	*L. amazonensis* (syn. *L. garnhami*)
			*L. mexicana*
		*L. tropica*	*L. aethiopica*
			*L. tropica*
	*L.* (*Viannia*)	*L. braziliensis*	*L. braziliensis*
			*L. peruviana*
		*L. guyanensis*	*L. guyanensis*
			*L. panamensis*
		*L. lainsoni*	*L. lainsoni*
		*L. naiffi*	*L. naiffi*
	*L.* (*Enriettii*)	*L. enriettii*	*L. siamensis* / *L. martiniquensis*

Only species present in the diagnostic records are included [11-13].

### Genomic targets and methods 

Several methods and genome loci were used for genomic typing purposes. These include sequencing, restriction fragment length polymorphism (RFLP) analysis, and/or subgenus-specific PCR of the following: heat-shock protein 70 gene (HSP70) [15-17], the internal transcribed spacer 1 of the rDNA locus (ITS1) [18,19], the mini-exon [19,20], multilocus sequence typing (MLST) genes [21], a repetitive sequence [22], and kDNA minicircles [14]. Both the target and method used were registered. Good quality sequences were submitted to GenBank.

### Ethical statement 

All data were shared and analysed anonymously in accordance with respective national guidelines. If needed, specific ethical clearance was obtained from the ethical committees or institutional review boards from the respective centres (ethical approval numbers are listed in the Supplementary Material: Ethics statements). 

## Results

**Figure 1:**
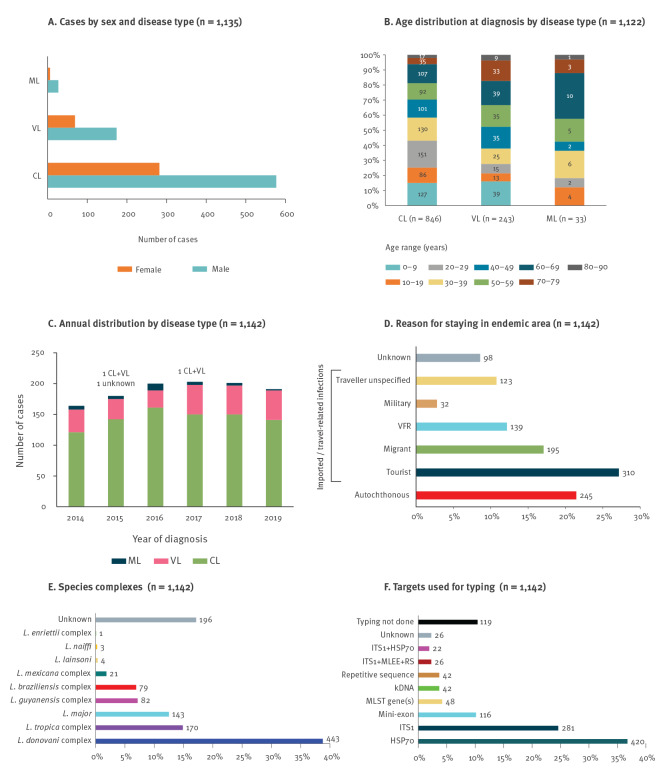
Overview of leishmaniasis cases identified by 15 European centres, 2014–2019

CL: cutaneous leishmaniasis; HSP70: heat-shock protein 70; ITS1: internal transcribed spacer 1 of the rDNA array; kDNA: kinetoplast DNA; ML: mucosal leishmaniasis; MLEE: multilocus enzyme electrophoresis [47]; MLST: multilocus sequence typing; RS: repetitive sequence; VFR: visiting friends or relatives; VL: visceral leishmaniasis.

Of the 1,142 records, more than half of cases were diagnosed in four countries: Italy (n = 232), France (n = 166), Sweden (n = 133), and Belgium (n = 124) (Table 1). Because some records had missing data, denominators mentioned throughout the text count only those records where data were available. Of all cases, 68% (776/1,133) were males and 32% (357/1,133) were females. Autochthonous infections represented 23% (245/1,044) of cases, while 77% (799/1,044) were travel-related. The median age at diagnosis was 37 years (range: 0–90), and 15% (166/1,120) were younger than 10 years. Numbers are stratified according to disease (Figures 1A and 1B).

Panel A and B: Two cases suffering concomitant VL and CL were counted in both categories. Panel B: Total case numbers depicted in each age range box. Panel D: ‘Traveller unspecified’ are imported cases for which the reason for travelling to an endemic area was unknown. ‘Unknown’ are either imported or autochthonous infections. Panel E: ‘Unknown’ was used where parasite typing was not done or was inconclusive. Panel F: Multilocus enzyme electrophoresis was performed according to reference [47]. ‘Unknown’ was used where the typing target was not recorded. The x axis for panels D-F shows percentages of all 1,142 records, the numbers on each bar are the actual values.

The distribution per annum during 2014–19 shows that the majority of cases were CL (76%; n = 865), followed by VL (21%; n = 241); only 3% (n = 33) showed mucosal involvement (Figure 1C). Two cases had concomitant CL and VL. In 1,044 cases, the reason for staying in an endemic area was classified (Figure 1D), with the majority being tourists (27%). The parasite was typed in 946 cases, most of which (47%) were infected with the *L. donovani* complex (Figure 1E). In 997 cases, the genomic target used for typing was recorded (Figure 1F), and 75% of these included the heat-shock protein 70 and/or internal transcribed spacer 1 of the rRNA gene array. In all samples typed with a recorded method (n = 950), sequence analysis was used in 74%, RFLP in 22%, and a subgenus-specific PCR in 4%.

The full dataset including GenBank accession can be downloaded and explored interactively in the MicroReact platform (https://microreact.org/project/leishman-2014-2019) [23].

### Country of infection

**Figure 2:**
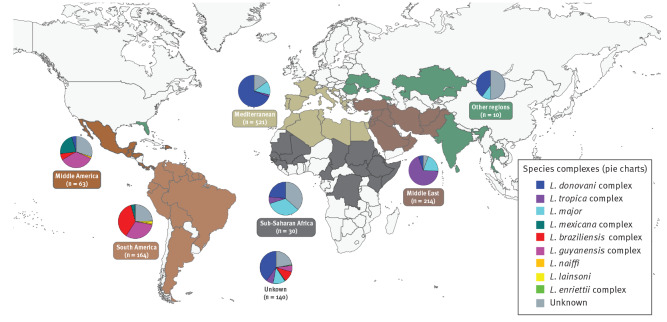
Probable region of infection and *Leishmania* species complex of leishmaniasis cases identified by 15 European centres, 2014–2019 (n = 1,142)

The probable country of infection was determined for 946 of 1,142 (83%) cases. These countries were pragmatically grouped into the endemic regions (Figure 2). Of the remaining 196 cases, 140 visited several of these endemic regions, and hence the origin of infection was impossible to establish. Finally, 56 cases visited different countries from the same endemic region, and for these the endemic region rather than the exact country is reported. The species complexes that were found in each of the regions, as well as the total caseload, are shown (Figure 2).

Each region is shown in a different colour (boxes) and the countries within each region where leishmaniasis cases were diagnosed are indicated on the map. The corresponding pie chart represents the *Leishmania* species complexes (Table 2) that were identified within each region (see legend). The number below the region name is the total number of cases represented in the respective pie chart. The category ‘Other regions’ (green) includes the countries or states not included in one of the five natural endemic regions. The pie chart ‘Unknown’ represents cases for which the origin of infection could not be linked to any of the six regions, either because the case visited several regions, or travel history was not known. These data can be interactively explored in detail at country level from the MicroReact platform (https://microreact.org/project/leishman-2014-2019).

Species from the *L. donovani* complex were found both in the New and Old World. Most of these were *L. infantum* (in the New World, also known as *L. chagasi*), while *L. donovani* was identified only from the Horn of Africa and Afghanistan. *L. tropica* was imported from northern Africa and the Middle East, with one case from Eritrea, while *L. aethiopica* was identified only from Ethiopia. *L. major* had a broad distribution in the Middle East and in Africa north of the equator. The *L. braziliensis* species complex was imported to Europe from Central and South America, with *L. peruviana* identified in one case from Peru. The *L. guyanensis* complex showed the same distribution, with *L. panamensis* originating only from the pacific coastline. *L. naiffi* was found in Belize and French Guiana. The *L. lainsoni* cases originated from Brazil and Peru. The *L. mexicana* complex was identified from several Latin American countries, with a marked dichotomy between *L. mexicana* in Middle America and *L. amazonensis* in South America. The *L.* (*enriettii*) species complex was identified in one case that visited several leishmaniasis endemic zones and could have been infected in either Guyana, Ghana, or Grenada in the Caribbean area.

### Subgroup results

**Figure 3:**
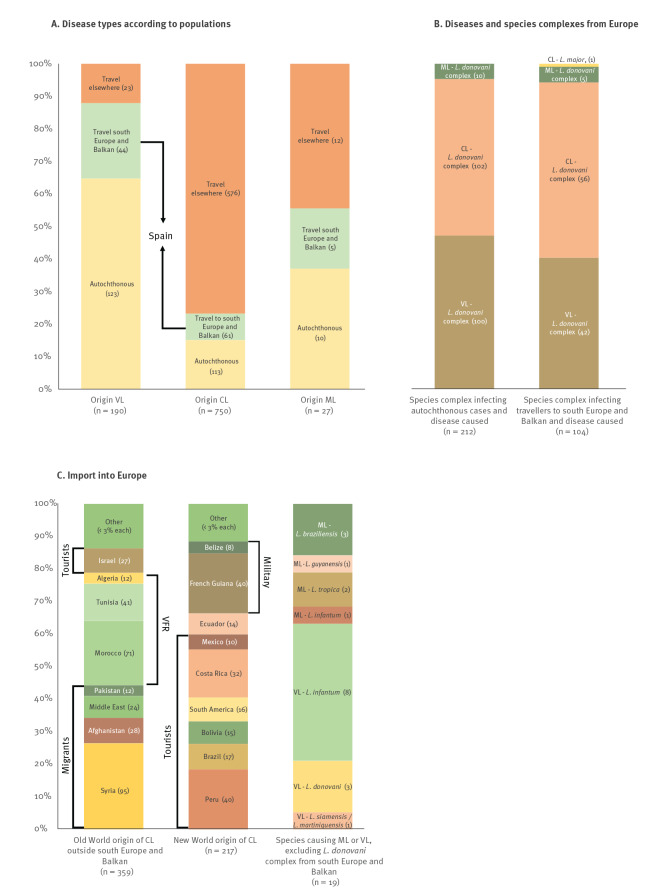
Stratification according to geographic origin, species complexes and populations of leishmaniasis cases from 15 European centres, 2014–2019

CL: cutaneous leishmaniasis; ML: mucosal leishmaniasis; VFR: cases who visited friends or relatives; VL: visceral leishmaniasis.

We examined relevant trends for different sub-categories of the variables (Figure 3). VL was primarily acquired within European countries (Figure 3A). Of the 190 VL cases, 65% were autochthonous infections and 23% originated from travels to southern European countries (mainly Spain) and the Balkan peninsula, while only 12% were infections acquired in other countries. In contrast, 77% of the 750 CL infections were imported from other countries, and thus only 23% were from south Europe and the Balkan region (Figure 3A). For the 27 ML cases, the difference in origin was not pronounced (Figure 3A).

The absolute case numbers follow the categories in each bar graph. The variables indicated alongside the bars represent over 50% of the cases in the bar categories, e.g. Panel C. over 50% of Old World CL from each individual country/area specified (Afghanistan, Pakistan, Syria, Middle East) are seen in migrants.

Of the 245 autochthonous cases, 212 were typed and found to be infected with a species from the *L. donovani* complex (Figure 3B), i.e. *L. infantum* in the 190 cases for which the species was determined. Five percent of these infections caused ML, the rest was equally divided over VL and CL pathologies. With the exception of one *L. major* infection causing CL, the same pattern was seen in travellers who were infected in southern Europe or the Balkan countries, but with slightly more CL than VL cases (Figure 3B). In this population, *L. infantum* was identified as the only species of the *L. donovani* complex.

Of the 576 CL cases with known origin imported into Europe, 62% and 38% were from the Old and New World, respectively. In the Old World (Figure 3C), mainly migrants contracted CL in Syria, Afghanistan, the Middle East in general, and Pakistan. VFR contracted CL in Morocco, Tunisia, and Algeria. In Israel, mainly tourists were infected and imported cases to Europe. For the New World (Figure 3C), tourists imported 53% of the CL cases to Europe, mostly from Peru, Costa Rica, Brazil, Bolivia, and Mexico. Travellers for whom the reason of travelling was not documented imported another 28% of cases, and several of these were probably tourists as well. Most infections from French Guiana and Belize were seen in military personnel.

All species complexes caused CL, except for the single *L. enriettii* species complex, which caused VL. As mentioned previously, *L. infantum* was the only species found to cause VL and ML in European-acquired infections. The parasite was typed to the species level in only 19 VL and ML cases infected outside Europe (Figure 3C). In these cases, VL was caused by *L. donovani*, *L. infantum* and *L. siamensis* or *L. martiniquensis*, while *L. braziliensis*, *L. tropica*, *L. guyanensis* and *L. infantum* caused ML.

## Discussion

We examined trends in autochthonous and imported leishmaniasis cases recorded by 15 centres throughout Europe between 2014–19 with respect to disease, age, sex, region/country of origin, species, and reason for travelling. Our analyses lend further support to previously described trends, with a specific focus on the European territory and integration of data from many centres in non-endemic countries. This adds to earlier reports describing a global picture of travellers, or focussing on European endemic countries only.

We observed a difference in age distribution between VL and CL, which is linked to the respective patient populations. Because CL is primarily imported into Europe, it tends to affect young active travellers: infant and adolescent migrants, military personnel, and tourists. In particular, young people who engage in activities with high risk of vector contact, e.g. backpacking and sleeping outdoors or in tents, are at increased risk. Further, children below 10 years of age can easily become infected when travelling to endemic regions, e.g. visiting friends or family, which can be explained by their immature immune system in combination with vector contact while playing outdoors. In VL, infants, children (< 10 years) and elderly people (> 60 years) were most often affected, which could be explained by the fact that VL is primarily an autochthonous disease caused by *L. infantum*, affecting people with a compromised immunity [24].

We observed that twice as many cases were male as opposed to female, both in VL and CL, which can be explained by two factors. First, in many parts of the world, males are more often engaged in outdoor activities and behaviour that could render them more prone to vector contact and thus infection and, second, females are less susceptible to severe disease [25].

We confirmed the findings of a literature review by Mansueto et al. [26] showing that VL cases diagnosed in Europe primarily originate from European endemic countries, and that cases are rarely imported from non-European regions. Also, we found *L. infantum* as the only causative species, confirming other reports [3]. The vast majority (> 60%) of cases, both with VL and CL, who were travellers to southern Europe were infected in Spain, which is in line with findings by Ehehalt et al. examining cases in European tourists during the period 2000–12 [4]. This reflects the popularity of the country as a holiday destination [27]. Boggild et al. [5] analysed 955 CL and ML cases in migrants and travellers reported in the global GeoSentinel surveillance network for the period 1997–2017, and they too identified Spain as one of the main source countries of leishmaniasis in travellers. Regarding domestic CL, a recent analysis from Greece on the number of CL vs VL cases during the period 2004–18 reported that only 2.7% (24/886) of domestic infections were CL [10]. This is a huge contrast with our analysis, where 55% (174/316) were CL. The reason for the difference is unclear.

Most CL cases were imported from outside the European territory. Boggild et al. found that Old World CL is imported primarily by migrants and VFR [5]. They identified Syria and Afghanistan as the main source countries for migrants who acquired CL, which is in line with our findings. This is not surprising, as migrants from these areas often emigrate to Europe [28]. This illustrates that a secondary effect of human migration from conflict zones – apart from the direct humanitarian crisis – is an increased risk of importing new pathogens into Europe. Only 10% of travel-related CL or ML cases were migrants according to Boggild et al. [5], while this number was 25% in our analysis. This can be attributed to the heavy caseload from Syrian and Afghan refugees in recent years. In both our analysis and that by Boggild et al., Tunisia, Morocco and Algeria are in the top five countries of acquisition for VFR, and both found that mainly tourists were infected in Israel.

In the Boggild et al. study [5], the three New World countries where most travellers acquired CL or ML were Bolivia, Costa Rica and Peru. Together with French Guiana and Brazil, these three countries made up the top five in our analysis; and as in [5] primarily tourists were infected. Of note, our findings show that Brazil, which covers half the area of the South American continent, represented only 8% of all New World CL cases.

Our analysis shows that among travellers, tourists may be at greatest risk for developing ML or VL, as 65% (166/254) were infected with species from the *L. donovani* or *L. braziliensis* complex. Where the species were identified, the latter were all *L. braziliensis* except for a single case of *L. peruviana*. The *L. donovani* complex species can cause both ML and VL, while *L. braziliensis* can lead to ML [6]. The risk of ML in tourists travelling to the New World was also identified in [5]. In addition, tourists and military personnel were infected with species from the *L. guyanensis* complex, occasionally causing ML as well [5]. However, only 5% (10/200) of tourists theoretically at risk based on these infecting species actually presented ML, and none (0/20) of the military personnel. From the Old World, we identified two migrants with ML in a total 124 infected with *L. tropica*, again indicating the limited risk. In contrast, of the 122 tourists infected with species from the *L. donovani* complex, 49 (40%) developed VL, underscoring a high risk.

Some of our typing records presented unexpected results in the context of the known epidemiology of species and disease distribution [29,30]. Cases of *L. infantum* were reported from Cameroon, Guinea, Kenya, India, the Dominican Republic, and the United States (US). In Cameroon and Guinea, *L. major* is the known aetiological agent of CL [29]. The *L. donovani* complex was previously isolated from a sandfly vector in Cameroon [31], but this is the first report of a CL and ML case. The case from Guinea had VL and, in this area, humans have been found to be seropositive both for *L. donovani* and *L. major* [32]. The few cases we identified provide further support to the spread of *L. infantum* in West Africa, as was previously evidenced from human VL and CL in various countries, the presence of and parasite isolation from the vector, and identification of leishmaniasis in dogs, typically considered the species’ reservoir [33]. In Kenya, different forms of CL were previously reported from *L. major*, *L. tropica*, and *L. aethiopica*, while VL is caused by *L. donovani* [29]. We detected a CL case caused by *L. infantum*, which is unique for East Africa. Even though *L. infantum* had been described based on multilocus microsatellite data, further analysis revealed they were all *L. donovani* [34,35]. Equally unique is the single VL case we report from an *L. infantum* infection acquired in India, while *L. donovani* is the causative agent in the country [29]. Evidently, no conclusions can be drawn from these single incidents, as we cannot rule out that the cases were infected in another country, and species typing was based on a single genome locus, i.e. a partial HSP70 sequence. Of note, microsatellite and whole genome analyses identified a separate clade in the *L. donovani* species complex, consisting of strains from Kenya and a subset of India and Ethiopia [34,36]. At present, we cannot exclude that these strains have an HSP70 sequence that would incorrectly classify them as *L. infantum.* Finally, autochthonous CL from *L. infantum* has never been documented in the Dominican Republic or the US, where two tourists were presumably infected. In both countries, autochthonous CL is sporadically caused by the *L. mexicana* complex [29,37,38]. In the US, canine leishmaniasis from *L. infantum* is common [29], but no human infections are known and no canine cases have been described in Florida, US [39], the only state visited by the case.

In Afghanistan, CL from *L. major* and *L. tropica* infections is common [29], but CL cases from the *L. donovani* complex have been documented in travellers and soldiers [40,41]. In our dataset, two CL cases in migrants infected with the *L. donovani* complex were identified, one of which was typed to the species level as *L. donovani*. Further, we counted three noteworthy CL cases caused by *L. major*, i.e. from Spain, Kazakhstan, and Ethiopia. Foci of *L. major* CL have been described in Kazakhstan, but the caseload is probably under-reported [29]. In Ethiopia, the species was found in bats and sandflies, but was so far not identified in humans [42]. In Spain, including the Balearic Islands [29], not a single case of an *L. major* infection has been described, and *L. infantum* is known as the only aetiological agent of leishmaniasis. The case in our database concerns a tourist who returned with many sandfly bites from the Balearic island of Ibiza, and did not travel to any other known place endemic for leishmaniasis. The species typing was confirmed twice using HSP70 sequencing. In addition, a migrant from Eritrea was diagnosed with the *L. tropica* complex, probably the *L. tropica* species rather than *L. aethiopica.* Little information on the species in this country is known, even though both VL and CL are present [29] and one report mentions an endemic *L. aethiopica* infection [43]. Finally, one *L. siamensis* / *L. martiniquensis* VL case in our dataset was described as originating from Guyana [44], but could have been infected elsewhere as this case visited Ghana and Caribbean Grenada, where this species was detected before [45]. Even though the aforementioned isolated cases do not imply local transmission in the respective countries, they may warrant species typing in patients returning from these areas.

### Limitations of the analysis

The main limitation of our study is that the 15 participating centres are from 11 countries in western Europe and Scandinavia, which generally do not capture all medically attended leishmaniasis cases in their respective countries. We estimate that in each of the 11 countries we overall reported between 10 and 100% of all imported infections, which is a representative sample in terms of species distribution, geographic origin, and type of travel. As is the case for all travel-related diseases, when and where a patient was infected often cannot be determined with absolute certainty, which highlights the need to pool data from many institutes to confirm individual extraordinary observations. As many CL manifestations are self-healing or can be efficiently treated in a peripheral dermatology practice, our data collection probably missed relatively more CL compared with VL cases, as happens even in countries where the diseases are notifiable [10].

When comparing our data to those of the World Health Organization (WHO) Global Observatory [46] (consulted on 26 Jul 2021), we may have systematically under-reported the proportion of autochthonous infections. Of the countries that are represented in our dataset, leishmaniasis is endemic in France, Italy, Spain and Portugal. For these countries, partial WHO data are available for the period 2014–18. Of all VL cases in these countries, 9% (52/557) were imported, while this was 18% (23/126) in our dataset for the same reference period and countries. For CL and ML together, 59% (619/1046) was imported according to WHO, while this was 64% (177/278) in our analysis. Even though data from WHO are incomplete and also biased, underestimation of autochthonous infections in our analysis would not be surprising, as typically autochthonous diseases are treated in many medical facilities without referral to the specialised centres participating in this study. It is also in line with centres from France, Spain and Portugal reporting few autochthonous infections. An alternative explanation for the discrepancy would of course be that WHO systematically under-reported imported cases.

## Conclusions

This study highlights the value of permanent monitoring of autochthonous and travel-related leishmaniasis cases in Europe. On one hand, such monitoring can confirm previous observations, on the other, surveillance can also draw attention to changes in the epidemiology of the diseases over time, or to endemic regions that have been under-studied and where awareness campaigns are needed. Cases imported into high-income countries, where effective species typing tools are available, are a rich source of information, provided that data from many medical centres are pooled to increase the number of observations. Resources like the WHO Global Health Observatory are excellent tools to implement such continuous surveillance. Since 2017, the Department of Control of Neglected Tropical Diseases of the WHO headquarters and the WHO Regional Office for Europe have provided financial and technical support to the European LeishMan network. With that support, databases have been aligned, allowing LeishMan centres to share their data of diagnosed leishmaniasis cases with WHO. This will improve surveillance of the epidemiology of the leishmaniases not only in Europe, but also in other parts of the world frequented by European citizens.

## MicroReact repository

Supplemental data view in the MicroReact [23] platform: https://microreact.org/project/leishman-2014-2019

Surveillance of diagnosed leishmaniasis cases in LeishMan centres over the period 2014–19. Data are shown at the country level, except for five pie charts located at the exact same positions as in Figure 2. These represent cases who were infected in one of five endemic regions (Mediterranean, Sub-Saharan Africa, Middle East, Middle America, South America) but for whom the exact country of infection could not be reliably established. The pie chart shown between South America and Africa represents the cases that could not be linked to any of the aforementioned leishmaniasis endemic regions.

## Supplementary Data

112000 Supplement
