# Supplementary material for: Surveillance of leishmaniasis cases from 15 European centres, 2014 to 2019: a retrospective analysis
Source: Euro Surveill. 2022 Jan 27;27(4):2002028. doi: 10.2807/1560-7917.ES.2022.27.4.2002028 (PMC8796293; doi:10.2807/1560-7917.ES.2022.27.4.2002028)
Supplement: Supplement [file 20-02028_AUWERA_Supplement.pdf]

Ethical approval numbers for anonymous data sharing of patient records

This supplementary material is hosted by Eurosurveillance as supporting information alongside the article [Surveillance of leishmaniasis cases from 15 European centres, 2014 to 2019: a retrospective analysis] on behalf of the authors who remain responsible for the accuracy and appropriateness of the content. The same standards for ethics, copyright, attributions and permissions as for the article apply. Eurosurveillance is not responsible for the maintenance of any links or email addresses provided therein.

Institute	Ethical committee	Approval reference
Institute of Tropical Medicine Antwerp	Institutional Review Board of the Institute of Tropical Medicine Antwerp	1095/16
Centre Hospitalier Universitaire de Rennes	Comité d'éthique du CHU de Rennes	n° 21.92
Necker Pasteur Paris	French National Agency	DR 2013DR-2013-041; N°912650
Charité-Universitätsmedizin Berlin	Data were anonymously analysed for surveillance, under the respective legal health regulations. Local regulations do not require formal approval	
INMI Lazzaro Spallanzani	IL COMITATO ETICO dell'Istituto Nazionale per le Malattie Infettive Lazzaro Spallanzani I.R.C.C.S.	PARERE N. 38/2016 del Registro delle Sperimentazioni
Istituto Superiore di Sanità Rome	Regional ethical board of Istituto Superiore di Sanità	RGPD 2016/679
University Hospital of Bologna	Ethical Committee of the St.Orsola-Malpighi University Hospital , Bologna, Italy	N.97/2017/O/Tess
Amsterdam University Medical Centres	MEC Leiden	P12.291
Elisabeth Tweesteden Hospital Tilburg	METC Leiden-Den Haag-Delft. (METC-LDD)	P12.291
Oslo University Hospital	Data were anonymously analysed for surveillance, under the respective legal health regulations. Local regulations do not require formal approval	
Instituto de Higiene e Medicina tropical	Ethical Commission of the Instituto de Higiene e Medicina Tropical	Parecer 9.21
Instituto de Salud Carlos III	Ethics Committee of the Hospital Universitario de Fuenlabrada	APR 12-67
Public Health Agency of Sweden	Regional ethical board of Lund	Dnr 2014/646
Swiss Tropical and Public Health Institute	EKNZ Ethikkommission Nordwest- und Zentralschweiz	EKNZ Req-2016-00050
	Ethikkomission beider Basel EKBB	303/11 and amendement 213/11
Hospital for Tropical Diseases	Data were anonymously analysed for surveillance, under the respective legal health regulations. Local regulations do not require formal approval	
